# Socio-demographic, environmental and caring risk factors for childhood drowning deaths in Bangladesh

**DOI:** 10.1186/s12887-015-0431-7

**Published:** 2015-09-10

**Authors:** Mosharaf Hossain, Kulanthayan K. C. Mani, Sherina Mohd Sidik, K. S. Hayati, A. K. M. Fazlur Rahman

**Affiliations:** Department of Community Health, Faculty of Medicine and Health Science, University Putra Malaysia, Serdang, Malaysia; Department of Psychiatry, Faculty of Medicine and Health Science, University Putra Malaysia, Serdang, Malaysia; Department of Epidemiology, Bangladesh University of Health Sciences, Dhaka, Bangladesh

**Keywords:** Social-demographic, Environmental and caring, Risk factors, Drowning, Bangladesh

## Abstract

**Background:**

Drowning contributes to incapacity and early death in many countries. In low- and middle-income countries, children are the most susceptible to fatalities. Over 50 % of the global drowning deaths occur among children aged under 15 years old with children aged between 1 and 4 years of age being most at risk. In Bangladesh, drowning rates are 10 to 20 times more than those in other developing countries. The object of this study is to determine the socio-demographic, environmental and caring hazard issues for child drowning in Bangladesh.

**Methods:**

A case–control study was conducted, with data collected from the Bangladesh Health and Injury Survey (BHIS) to identify the social-demographic and environmental factors associated with childhood drowning. The participants represented 171,366 households from seven divisions of Bangladesh—Dhaka, Rajshahi, Chittagong, Barisal, Sylhet, Khulna and Rangpur. The survey was conducted between January and December of 2003. A total of 141 children drowning were identified in the year preceding the survey. Data were analysed using descriptive statistics and logistic regression analysis. The odds ratios with 95 % CI intervals were estimated for various associated factors for child drowning deaths.

**Results:**

In Bangladesh, in 2003, the incidence of drowning deaths was 104.8 per 100,000 among those aged less than 5 years; 168.7 per 100,000 in rural areas; male 32.4 per 100,000; 112.7 per 100,000 between 10:00 a.m. and 2:00 p.m.; and cannot swim 134.9 per 100,000. The socio-demographic danger factors for child drowning deaths were: being male (OR = 1.45, 95 % CI = 1.34–1.78), aged less than 5 years (OR = 2.89, 95 % CI = 1.89–3.11), urban areas (OR = 0.67, 95 % CI = 0.67–1.87), and mother being illiterate (OR = 1.69, 95 % CI = 1.01–2.81). Significant environmental and caring factors included mother/caregiver not being the accompanying person (OR = 25.4, 95 % CI = 14.4–45.3) and children cannot swim (OR = 4.5, 95 % CI = 1.25–19.4).

**Conclusion:**

Drowning is the single largest reason for the mortality of children aged less than five years. There is a need to educate Bangladeshi parents and encourage behavioural change concerning supervision. The Government should use mass media to raise awareness about drowning among the community with a focus on rural areas. Policies should focus on increasing supervision by mothers/care persons, swimming skills, and should target illiterate mothers. Therefore, there is an immediate need for the Bangladeshi Government to address the problem of drowning.

## Background

The classification of drowning is that accepted by the first world assembly on drowning: the process of experiencing respiratory impairment from submersion or immersion in liquid; outcomes are classified as mortality, illness and no morbidity [1]. Drowning is the third highest reason for accidental injury globally, and 7 % of all injuries connected with death worldwide are caused by drowning [2]. An estimated per year 372,000 drowning deaths occurred in the world [2]. Among children, drowning accounts for a higher mortality rate than any other type of child injury, and the drowning rate in Asia is 20 times higher than in developed countries [3]. Who is at risk? Children under 5 years of age, males, people exposed to water, those with low socio-economic status or lacking education, and infants left unsupervised [2]. Drowning is the single highest cause of mortality after infancy: 50 % of drownings occur between the ages of 0 and 4 years; 60 % of drownings occur between 9:00 a.m. and 1:00 p.m.; the majority (80 %) of drowning mortality happens in natural water bodies like millponds and channels; the maximum (80 %) occur within 20 cm of the family; and children of large households are more in danger of drowning than those from small households [4]. In Bangladesh, every half an hour a child drowns, this is 50 drowning deaths a day and 18,225 drowning deaths per annum [4].

Bangladesh is a mainly pastoral low-income country with the majority of homes situated near bodies of water [5]. Many children are unable to swim and families are large, making adult supervision difficult [6]. These issues contribute to make drowning the foremost reason of child mortality after infancy in Bangladesh. The drowning death rate, 28.6 per 100,000 child-years, is 22 times larger than in the Americas [5–10]. The virtual death due to underwater deaths in children was about 28 % for common child death [11]. Ponds, ditches, buckets and drums were the most common spaces for drowning, and over 40 % happened in millponds [11]. The child’s gender, mother’s age and literacy, family income and ownership of agricultural land by the families were found to be risk factors [11]. The occurrence of drowning among children aged under five years was 157 per 100,000 children-year [12]. The highest rate by single year of age and gender was 328.1 per 100,000 (95 % CI 254.8–421.7) for 1 year old male children [13]. The proportional mortality due to drowning in the children was 27.9 % for overall child mortality. The risk factors were mother’s age, literacy, family income. Male children from deprived household were at greatest risk of drowning in rural Bangladesh [14].

In 2015, in Bangladesh, children aged between 1 and 17 years injury was the main reason of death and Drowning is the highest [15]. The significant advancements in the decrease of child deaths from communicable infection in Bangladesh over the last two years has meant that injuries represent a greater danger to children [15]. Although the rates of drowning have been constant since the 1980s, drowning as a percentage of all types of mortality in children aged 1–4 has increased from 9 % in 1983 to 59 % in 2003 [15]. The purpose of the Millennium Development Goal (MDG 4) is to reduce child deaths. The Bangladesh Government target is to reduce the deaths of children under five years of age by two-thirds, and, overall, by 48 per 1000 by 2015 [15]. The Government expends a lot of money on HIV/AIDS through advertisements on the TV, radio and billboards, as well as in newspapers, but less attention is paid to the problem of drowning, which is one of the significant issues in the society. The objective of this study is to define the socio-demographic, environmental and caring risk factors concerning drowning in Bangladesh.

## Methods

### Study design

A case–control study was conducted as a separate component of the Bangladesh Health and Injury Survey (BHIS) to determine the socio-demographic, environmental and caring risk factors on drowning in Bangladesh.

### Sample size

The Bangladesh Health and Injury Survey (BHIS) is the leading injury investigation directed at the public level in a developing country, with a trial scope of 171,366 homes and a total piloted people of 819,429. The investigation was piloted between 1 January and 30 December 2003, and included all age groups; 43 % (351,651) of the surveyed population were children, and data for children under five were collected from parents. Children were defined in this report as infants and children of all ages, up to their 18th birthday (0–17 years old). A total of 141 child drowning deaths was identified in the year preceding the survey. When a child drowning death was discovered during the interview process, controls were selected and interviews conducted by the same interviewer who identified the case.

### Data collection

The field activities for data collection were conducted between January and December 2003 by BHIS. The BHIS is the main injury investigation directed at the public level in a developing country.

### Sampling technique

Twelve out of 64 districts were randomly selected for the survey in Bangladesh. In each region, one upazila (rural sub-region) was randomly nominated. In each upazila, two unions (lowermost governmental parts collected of almost 20000 persons) were nominated randomly. Overall, 24 unions were nominated for the study. All families in the union were included in the investigation.

### Respondents

Mothers (mothers were surveyed on behalf of their children) were preferred as the main respondent. When a mother was unavailable, the most well-informed member (uncle, grandfather or grandmother) of the family members present at the time of conversation was the participant.

### Cases

Drowning deaths between 1 January and December 2003.

### Controls

Age and sex were matched per one child drowning to at least two living children.

### Data analysis

The numerical examination of the results was slow using the IBM SPSS statistics Version 21. Data were analysed using descriptive statistics and logistic regression analysis. Logistic regression was used to determine the danger issues with children drowning mortality and respondent’s age, place of home, gender, mother’s learning level, father’s educational level, mother’s occupation, father’s occupation, number of children, accompanying person and swimming ability. The 95 % CI (Confidence Interval) level was measured significant at a P value of 0.05. The dependent variable was used the two dichotomous: Y = 1 if have child drowning deaths and Y = 0, otherwise. Respondent’s age, place of residence, gender, mother’s educational level, father’s educational level, mother’s occupation, father’s occupation, number of children, accompanying person and swimming ability were measured as forecaster variables in this classical.

### Ethical issues

Written or verbal knowledgeable agreement was obtained from all the respondents before collecting data. The respondents were assured that the data would only be used for research purposes and that all answers would be treated as confidential. Permission for this study was obtained from the Ethical Review Committee of the Ministry of Health and Family Welfare, Dhaka, Bangladesh.

## Results

Drowning deaths peaked in the 0–4 age group (104.8 per 100,000) and then rapidly declined as age increased: 5–9 years 26.2 per 100,000, 10–14 years 2.9 per 100,000 and 15–17 years 2 per 100,000. The near drowning was highest the children aged 0–4 years 443.7 per 100,000 and then decreased near drowning with increased age. Significantly different drowning rates were observed among different age groups of rural and urban children in Bangladesh. Urban areas have low rates, whereas those in rural areas are higher. Children under five years in pastoral areas had the highest drowning rate (136.9/100,000). The age groups 10–14 and 15–17 did not appear as the drowning deaths in these age groups were too low to enable a meaningful comparison between urban and rural distributions. There is a significant dissimilarity in the rate between males and females in the same age group. Males are at greater risk of drowning. The drowning death rate for children 1–17 years was 28.6/100,000, of which males were 32.4/100,000 and females 24.8/100,000. Almost all (97 %) drownings occurred during the hours of daylight between 10:00 a.m. and 2:00 p.m. Other factors include the swimming ability of children aged over 4 years who drowned and the person accompanying the children prior to drowning (Tables 1 and 2).

**Table 1:** Child drowning deaths rates and near drowning rates by social-demographic, environmental and caring factors

Factors	Rates (Per 100000)
Child drowning deaths rates by age (Years)	
0–4	104.8
5–9	26.2
10–14	2.9
15–17	2.0
Child near drowning rates by age (Years)	
0–4	443.7
5–9	73.9
10–14	13.6
15–17	4.8
Place of residence	
Rural	168.7
Urban	33.4
Gender	
Male	32.4
Female	24.8
Time when children to drowning deaths	
10:00 AM–2:00 PM	112.7
Others time	34.6
Accompanying person	
Mother/Caregiver	45.9
Others	88.7
Swimming ability	
Can swim	5.7
Cannot swim	134.9

**Table 2:** Child drowning deaths rates (per 100000) in Bangladesh

Factors	Rates (Per 100000)	
Age (Years)	Urban	Rural
0–4	33.1	158.7
5–9	10.8	38.9
10–17	2.9	10.8
Age (Years)	Male	Female
0–4	113.9	95.2
5–9	31.1	21.0
10–17	9.6	1.0

Child age is the major risk factor for drowning deaths. Children aged 0–4 years were at 2.89 (OR = 2.89, 95 % CI = 1.89–3.11) times higher risk of drowning compared to children aged 10–17 (reference group) years, while children aged 5–9 years were at 1.66 (OR = 1.66, 95 % CI = 1.44–1.98) times higher risk of drowning deaths than the reference group. Males were at 1.45 times increased risk of dying from drowning compared to females. Place of residence is also a risk factor for childhood drowning deaths. For urban children it is 0.67 (OR = 0.67, 95 % CI = 0.34–1.87) times the risk of that of rural children. The results indicated that males are at 1.45 times higher risk than females (OR = 1.45, 95 % CI = 1.34–1.78). The results showed that having knowledge and awareness have a statistically significant effect on drowning deaths. The proportion of illiterate mothers in child drowning cases is 0.34, while the proportion of educated mothers in child drowning cases is 0.20. A child with an illiterate mother has a 1.7 (OR = 1.7, 95 % CI = 1.01–2.81) increased risk of drowning compared to a child whose mother has secondary education. A child of a mother who has five or more children in the family is 1.95 times more risk of drowning than a child of a mother who has less than 3 (OR = 1.95, 95 % CI = 1.2–3.3) children. When children drowned the accompanying persons was alone (OR = 25.4, 95 % CI = 14.4–45.3) 25 times risk for drowning to compare the childrens accompanying persons was others relative such as grandmother, grandfather, uncle, etc. Absence of swimming skills (for children 5–17 years) was associated with childhood drowning deaths and children who cannot swim are 4.5 times more at risk than children who can swim (OR = 4.5, 95 % CI = 1.25–19.4) (Table 3).

**Table 3:** Risk factors between social-demographic, environmental and caring factors with childhood drowning deaths, BHIS, 2005

Factors	Cases		Control		Odd ratio	95 % CI
	Number	Percentage	Number	Percentage		
Children’s age (Years)						
0–4	105	74.6	145	42.7	2.89^*^	1.89–3.11
5–9	25	17.7	102	30.1	1.66^*^	1.44–1.98
10–17 (RC)	11	7.7	92	27.2	1.00	……….
Gender						
Male	80	56.7	178	52.5	1.45^*^	1.34–1.78
Female (RC)	61	43.3	161	47.5	1.00	………
Place of residence						
Urban	39	27.6	198	58.4	0.67^*^	0.34–1.87
Rural (RC)	102	72.4	141	41.6	1.00	……..
Mothers Education						
Illiterate	70	49.6	135	39.8	1.69^*^	1.01–2.81
Primary	43	30.5	113	33.3	1.24	0.71–2.14
Secondary + (RC)	28	19.8	91	26.8	1.00	……….
Fathers education						
Illiterate	64	45.4	139	41.0	1.10	0.68–1.77
Primary	30	21.3	88	26.0	0.81	0.46–1.46
Secondary + (RC)	47	33.4	112	33.0	1.00	……..
Mothers occupation						
Home maker	135	95.7	326	96.2	1.00	………
Other than housewife	6	4.3	13	3.8	1.11	0.37–3.23
Fathers occupation						
Cultivator (RC)	98	69.5	221	65.2	1.00	………
Other occupation	43	30.5	118	34.8	0.82	0.53–1.28
Mothers age						
25–29 years (RC)	47	33.3	117	34.5	1.00	………..
Less than 20 years	4	2.8	9	2.7	1.11	0.27–4.20
20–24 years	21	14.9	76	22.4	0.69	0.37–1.29
30 years+	69	48.9	137	40.4	1.25	0.78–2.01
Number in children						
Less than 3 (RC)	45	31.9	137	40.4	1.00	…….
3–4	46	32.6	124	36.6	1.13	0.68–1.87
5 & more	50	35.5	78	23.0	1.95^*^	1.2–3.30
Accompanying person						
Mother/Caregiver (RC)	21	14.9	276	81.7	1.00	……..
Others	120	85.1	62	18.3	25.4^*^	14.4–45.3
Swimming ability			26	7.7	1.00	…….
Can swim (RC)			142	42.1	4.5^*^	1.25–19.4
Cannot swim						
Model summary:						
2 log likelihood: 242.922						
Cox & Snell R Square: 0.51						
Negelikereke R: 0.60						
Model Chi-Square: 8.019						

*RC* = Reference category, *p = Level of significant, Source: BHIS, 2005

## Discussion

Bangladesh has made great progress in addressing the issue of communicable diseases, which affects the mortality of infants and children under-five. Age is a major issue in terms of drowning mortality. The highest rate of drowning deaths was identified in the under five year age group (104.8 per 100,000 for fatal and also 443.7 per 100,000 for non-fatal). The age shape of drowning deaths was identical that, only the rates drowning deaths were several times higher (118/100,000 near drowning; 28.6/100,000 drowning) among children aged 1–17 years [16]. In Bangladesh the mean percentage of drowning deaths for all causes of injury deaths was 28 % [17]. The maximum drowning deaths were between male was 32.4 per 100,000 and 24.8 per 100,000 for female. The mother’s lack of education, as well as infants left alone, children under 5 years of age, especially males, are the factors that represent the greatest risk from drowning; this is consistent with other studies [3]. The drowning rate was highest for those younger than 5 years old, which is consistent with drowning data from other parts of the world [18, 19]. However, males aged 65 years and older had higher rates than females in Australia [20], while another study showed that, in Australia, 76.4 drowning deaths were male, with children aged under five years having the highest rate (2.63 per 100,000 persons) [21]. However, in Bangladesh, underwater deaths is the single highest cause of death after infancy; 50 % of drowning deaths occur between the ages of 0 and 4 years [4]. The lack of swimming ability was the highest cause of drowning among the children.

Males had greater underwater death rates compared to females. Our outcomes have similarity with these studies. In New Zealand, 76 % of unintentional drownings involved males [22]. The highest number of underwater deaths occurred among males in Louisiana with 84 % [23]. An assessment of Peden and McGee [19] exposed that males had a higher death rate due to drowning compared to females for all age groups. Lindholm and Steensberg [24] stated that, in Denmark, males were involved in 72.5 % of underwater deaths. The underwater deaths involving males in Iran was 87 % and the ratio of male–female mortality rate was 6.5:1. The outcome of Ma et al. [25] showed that boys were at greater danger of drowning than girls in China. In the United States, the percentage of mortality was meaningfully superior among males than among females. Overall, males are more likely to drown than females. The outcomes showed that males were particularly at danger of drowning in Bangladesh.

Drowning prevention programmes should include active prevention, such as establishing child care at home, especially for children under five years old; increase knowledge and awareness about childhood drowning, especially among rural parents; aquatic protection supervision by close relative; aquatic security teaching; drowning education for children in every school; and fixing of cautionary symbols in dangerous areas in Bangladesh. The current research studied drowning deaths in Bangladesh based on the available data in terms of gender and age group. Study limitations include the fact that the majority drowning cases go unrecorded. The next restriction is the absence of evidence from hospital and clinics, and the final limitation is information bias, as original research is costly and this study was dependent on sources of data from others, such as police data and post-mortem data.

## Conclusion

Age is the main contributory factor for drowning deaths. Other factors include gender (male), place of residence (rural area children), time of day (10:00 AM-2:00 PM), and parents educational level. The lack of attention of parents concerning their children and the lack of proper supervision of children swimming of (five years and over) contribute to the high number of drownings. Most of the Bangladeshi community has open ponds/rivers without boundaries and protection, which allows free access for drowning. Due to the magnitude of the drowning problem the government should develop and deliver an integrated programme via the media, education, ponds should be fenced, and skills should be developed to reduce drowning. Policies should focus on increasing supervision by mothers/care persons, swimming skills, and should target illiterate mothers. Therefore, there is an immediate need for the Bangladeshi Government to address the drowning problem.
